# Improving U-net network for semantic segmentation of corns and weeds during corn seedling stage in field

**DOI:** 10.3389/fpls.2024.1344958

**Published:** 2024-02-09

**Authors:** Jiapeng Cui, Feng Tan, Nan Bai, Yaping Fu

**Affiliations:** ^1^ College of Engineering, Heilongjiang Bayi Agricultural University, Daqing, China; ^2^ College of Electrical and Information, Heilongjiang Bayi Agricultural University, Daqing, China; ^3^ Heilongjiang Academy of Agricultural Mechanization Sciences, Suihua, China; ^4^ Soybean Research Institute, Heilongjiang Academy of Agricultural Sciences, Harbin, China

**Keywords:** smart weeding equipment, grass seedling identification, semantic segmentation, computer vision, precision agricultural

## Abstract

**Introduction:**

Weeds are one of the main factors affecting crop growth, making weed control a pressing global problem. In recent years, interest in intelligent mechanical weed-control equipment has been growing.

**Methods:**

We propose a semantic segmentation network, RDS_Unet, based on corn seedling fields built upon an improved U-net network. This network accurately recognizes weeds even under complex environmental conditions, facilitating the use of mechanical weeding equipment for reducing weed density. Our research utilized field-grown maize seedlings and accompanying weeds in expansive fields. We integrated the U-net semantic segmentation network, employing ResNeXt-50 for feature extraction in the encoder stage. In the decoder phase, Layer 1 uses deformable convolution with adaptive offsets, replacing traditional convolution. Furthermore, concurrent spatial and channel squeeze and excitation is incorporated after ordinary convolutional layers in Layers 2, 3, and 4.

**Results:**

Compared with existing classical semantic segmentation models such as U-net, Pspnet, and DeeplabV3, our model demonstrated superior performance on our specially constructed seedling grass semantic segmentation dataset, CGSSD, during the maize seedling stage. The Q6mean intersection over union (MIoU), precision, and recall of this network are 82.36%, 91.36%, and 89.45%, respectively. Compared to those of the original network, the proposed network achieves improvements of 5.91, 3.50, and 5.49 percentage points in the MIoU, precision, and recall, respectively. The detection speed is 12.6 frames per second. In addition, ablation experiments further confirmed the impactful contribution of each improvement component on the overall semantic segmentation performance.

**Discussion:**

This study provides theoretical and technical support for the automated operation of intelligent mechanical weeding devices.

## 1 Introduction

Weed control presents a significant challenge in agricultural production (Guo et al., 2018; Marchioro and Krechemer, 2018; Horvath et al., 2023). In this context, weeds such as dandelions, nettles, and pigweeds are typically characterized as wild plants growing in undesired locations. Since they rapidly grow and compete with crops for essential resources such as water, sunlight, and nutrients, they considerably hamper crop yields (da Conceição de Matos et al., 2019; Moreau et al., 2022). Presently, the primary methods for weed control are chemical and mechanical (Machleb et al., 2020; Zhao et al., 2023). Chemical weed control relies on agrochemicals to control weeds. However, this approach not only introduces environmental side effects but also leads to certain weeds developing resistance to these pesticides, making them difficult to eliminate. The widespread adoption of herbicides has led to a concurrent increase in weed resistance in fields. By contrast, mechanical weeding offers advantages such as lower labor costs, reduced environmental pollution and improved work efficiency. However, due to the irregular growth patterns of weeds in diverse environments, mechanical weeding machinery often struggles to distinguish between weeds and crops, potentially harming crops during this process. Therefore, accurate identification of weeds is pivotal in the mechanical weeding process. Therefore, devising a system through the application of advanced artificial intelligence technology for the automated identification of crops and weeds holds positive significance for improving weeding efficiency and reducing crop damage (Raja et al., 2020; Wu et al., 2020; Rai et al., 2023).

In recent years, with the widespread application of artificial intelligence technology in the agricultural sector (Ma et al., 2023; Wang et al., 2023; Yang et al., 2023), researchers have actively engaged in studies related to weed and seedling identification (Wang et al., 2019; Ashraf and Khan, 2020; Coleman et al., 2022). These studies primarily rely on advanced technologies in machine learning and computer vision. In weed identification methods based on machine learning, image processing techniques must be integrated, including the extraction and preprocessing of features such as color, shape, and texture (Ruslan et al., 2022). Alhwaiti et al. (2023) proposed an image analysis method for weed classification to support early weed detection in agriculture. To mitigate the influence of environmental factors, they introduced homomorphic filtering for preprocessing. For feature extraction, they innovatively introduced an adaptive feature extraction method that accurately estimated the direction of edges using edge detection and non-maximum suppression. This method has the advantages of user friendliness and scalability. Ultimately, through logistic regression to evaluate the extracted features, the weighted average recognition rate reached an outstanding 98.5% in natural environments, providing robust support for the further development of weed classification systems. Parra et al. (2020) presented a weed recognition method based on edge detection technology, aiming to reduce pesticide usage by early detection of invasive plants. They applied 12 edge detection filters to small experimental plots inside IMIDRA facilities and golf course images, representing ornamental lawns and sports lawns, respectively. Through in-depth analysis, they identified the sharpening (I) filter and minimum aggregation technique as optimal, with a unit size of 10, for achieving the best results. Finally, by adjusting the threshold to meet the evaluation metrics (precision, recall, and F1 score), 78 was confirmed to be the optimal threshold. This study provides an effective and environmentally friendly detection method for early weed identification. Although these weed recognition methods based on traditional machine learning have achieved good identification results, feature extraction is laborious and susceptible to various factors. Hence, they face challenges in expressing the complete content of images, leading to severe constraints on the generalizability, real-time performance, and repeatability of such recognition methods (Chen et al., 2020). To address the limitations of machine learning methods in weed recognition tasks, researchers are extensively exploring computer vision-based approaches. This method can automatically learn and extract complex features from images, effectively overcoming the constraints of manually designed features and achieving a more comprehensive understanding of images. This approach significantly enhances the accuracy and real-time performance of weed recognition, allowing it to reach state-of-the-art levels of performance (Hu et al., 2024). Computer vision-based approaches can be further subdivided into subtasks such as image classification, object detection, and semantic segmentation. This segmentation allows us to address different recognition problems more effectively. Image classification helps to accurately identify weed categories in images (Hasan et al., 2023); object detection can discover and locate multiple instances of weeds in images, while semantic segmentation provides a detailed division of weeds and backgrounds at the pixel level (Wu H. et al., 2023). By employing these computer vision methods, we can handle complex scenarios more flexibly and capture details in images more accurately (Veeragandham and Santhi, 2024). This approach not only contributes to enhancing the overall performance of weed recognition but also lays a solid foundation for further improvement and development of related technologies (Hu et al., 2024). In the task of weed image classification, Wu Y. et al. (2023) proposed a method that integrates a convolutional neural network (CNN) and a support vector machine (SVM) to train and test the DeepWeeds public multiclass weed dataset. The proposed fusion methods, ResNet-50-SVM and VGG16-SVM, achieved the best accuracy compared to the contrasted CNN methods VGG16, ResNet-50, GoogLeNet, DenseNet-121, and PSO-CNN. However, due to the use of SVM as a classifier, the model’s runtime is relatively slow. Mu et al. (2023) introduced an ECA attention mechanism into a DenseNet weed classification model. Through comparative experiments with DenseNet, VGGNet-16, VGGNet-19, and ResNet-50 on processed weed image datasets, the improved model achieved the best accuracy. However, this model solely classifies weeds and does not consider crop and soil information. In the task of weed detection, Zhang et al. (2022) proposed an improved Faster-RCNN algorithm for corn seedling and weed recognition. They compared VGG16 and ResNet as feature extraction networks and ultimately chose VGG16 to implement Faster-RCNN for corn and weed recognition. However, this method has a slow recognition speed, making it challenging to meet real-time recognition needs. Pei et al. (2022) used an improved YOLOv4 model as a weed detection model, introducing the Meta-ACON activation function, adding the convolutional block attention module (CBAM), and replacing non-maximum suppression (NMS) with soft non-maximum suppression (Soft-NMS). However, this approach struggles with detection when corn seedlings and weeds overlap or occlude. In the task of weed semantic segmentation, Guo et al. (2023) utilized a vision transformer (ViT) as the feature extraction network for a U-net semantic segmentation network. They used the Python Imaging Library algorithm to segment green plant leaves from binary images, enhancing feature extraction. This improved method demonstrated high accuracy in corn seedling and weed recognition. However, ViT is prone to overfitting on small datasets, making the matching of model complexity and data volume important considerations. Mu et al. (2022) proposed a recognition method based on an improved Faster R-CNN using ResNeXt as the feature extraction network and an integrated feature pyramid network (FPN) for feature pyramid fusion. Although fusing information from different scales can improve recognition accuracy, it also increases the computational complexity of the model. Therefore, real-time segmentation is challenging in this two-stage semantic segmentation model. Zhang et al. (2023) presented a weed recognition model based on an improved Swin-Unet model. The model initially adopts U-Net as the semantic segmentation framework, introduces the Swin Transformer module to enhance performance, and finally uses an improved morphological processing algorithm to identify and segment weedy regions. Comparative experiments with DeepLabv3+, PSANet, and Mask R-CNN on a weed dataset showed that the improved Swin-Unet model achieved optimal performance, demonstrating good adaptability of the encoder-decoder structure semantic segmentation network for weed recognition. Liu et al. (2023) proposed an improved multi-scale efficient residual factorized network (MS-ERFNet) model for identifying corn seedling rows. In comparative experiments with DeepLabv3+, ENet, ERFNet, FCN-8s, U-Net, and MS-ERFNet on a corn seedling row test set, the average intersection over union and pixel accuracy were greater for the MS-ERFNet model than for the other models. This finding suggested that semantic segmentation is a suitable seedling recognition method for effectively alleviating the issue of reduced recognition accuracy due to mutual occlusion between seedlings. In summary, deep learning-based detection methods have become the dominant approach in the field of weed recognition (Hu et al., 2024). Through automatic learning, deep learning can extract the required features from data, effectively overcoming the limitations of manual feature engineering methods. In weed recognition tasks, we face various challenges, such as diversity in the size and shape of weeds, mutual occlusion between weeds and crops, and the identification of densely distributed targets. To address these challenges, an increasing number of researchers are attempting to apply semantic segmentation algorithms in deep learning for weed recognition. Semantic segmentation algorithms based on deep learning have the advantage of pixelwise classification, enabling effective segmentation of irregularly shaped and densely distributed objects. However, current semantic segmentation models for weed recognition face several challenges. These include the complexity of recognition models, difficulty in ensuring real-time performance, limited ability to handle overlapping occlusions, and the need for improvement in recognition accuracy.

Aiming at the problems existing in seedling grass recognition within maize seedling fields, we propose an improved semantic segmentation network, RDS_UNet, based on the U-net network. This enhanced model demonstrated superior semantic segmentation performance on our self-constructed seedling grass semantic segmentation dataset, CGSSD, which was specifically tailored for maize seedling fields. By doing so, we aim to support seedling grass recognition in intelligent precision weeding operations. Our main contributions include the following three aspects:

1.ResNeXt-50 was adopted for feature extraction by the encoder. The integration of residual and inception structures within ResNeXt ensures the mitigation of vanishing gradient issues and obtains better feature extraction at lower computational costs.
2.In the decoder’s Layer 1, traditional convolution is supplanted by deformable convolution, which is equipped with learnable offsets. This deformable convolution increases the receptive field, improves the pixel correlation for segmentation, and increases the robustness of the model.
3.After standard convolution in the decoder, layers 2, 3, and 4 were enriched with the concurrent spatial and channel squeeze & excitation (scSE) hybrid attention mechanism. This hybrid attention mechanism—channel and spatial—effectively curtails superfluous features, thereby improving semantic segmentation accuracy.

## 2 Materials and methods

### 2.1 Construction of semantic segmentation dataset for seedling grasses in the field during seedling stage of maize

#### 2.1.1 Data acquisition

**Figure 1:**
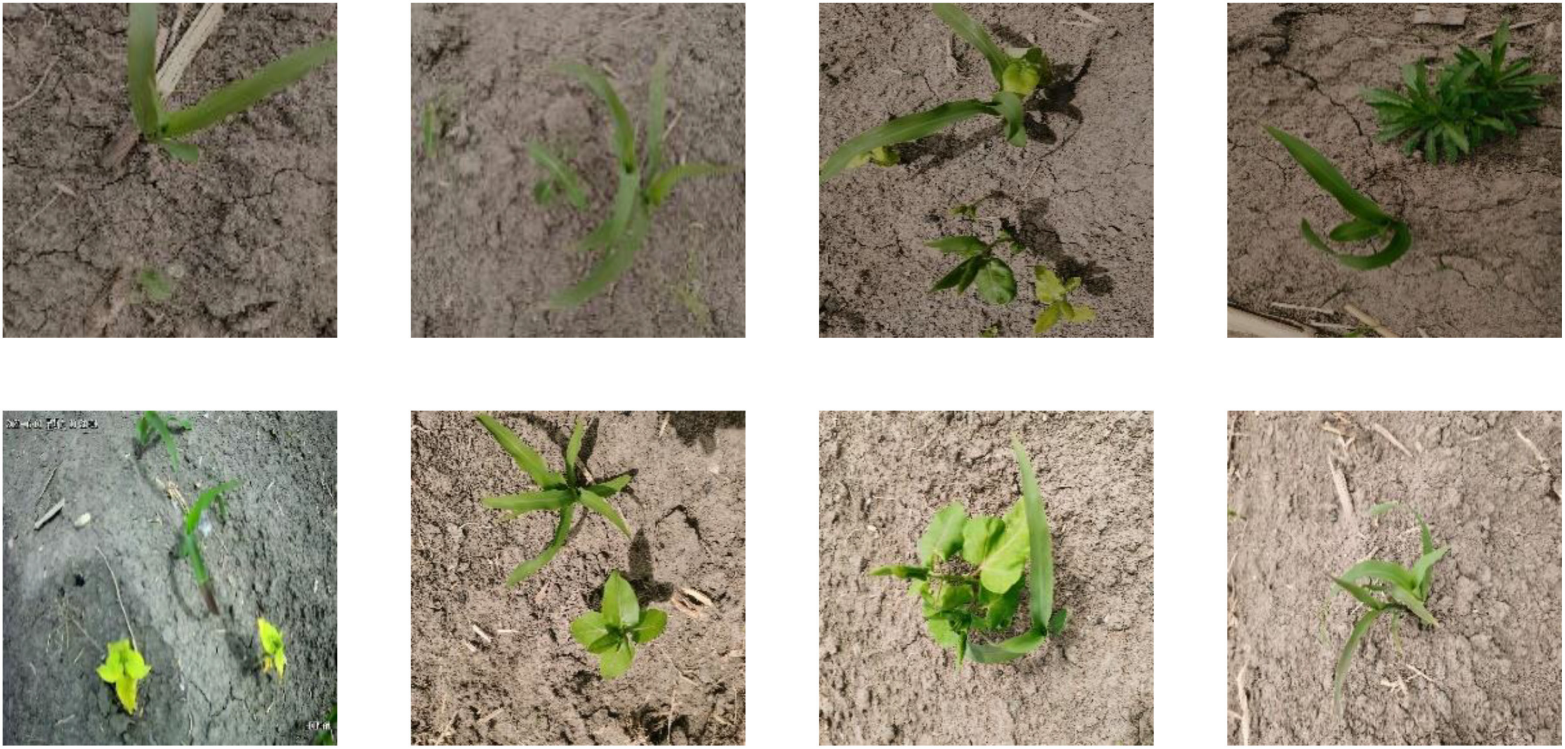
Example of a CGSSD data sample.

Our study focused on maize plants and their associated weeds. Between May 10th and 20th, 2023, experimental images were collected multiple times from the Heilongjiang Bayi Agricultural Reclamation University’s experimental base located in Anda city, Heilongjiang Province. The images were captured using a YH-IPC-U 33 WH camera equipped with a 3MP definition, a 1/3 inch photosensitive area, a 6 mm focal length, a horizontal rotation spanning 0°–355°, and a vertical rotation from -5°–90°. Positioned vertically 0.8 m above the ground, this ball machine camera recorded videos with a resolution of 1280 × 720 pixels. These videos were then segmented into images of the same resolution in JPEG format. After removing consecutive frames containing the same corn plants and their associated weeds, 500 sample images containing corn plants and their companion weeds (including grassy and broadleaf weeds) were manually selected by agricultural experts. These sample images encompass various weed sizes, weed densities, diverse weed phenotypic information, and typical instances of seedling overlap and occlusion. Postprocessing involved adjusting these images to 224 × 224 pixels. Subsequently, annotations were made using Labelme, with “corn” indicating maize seedlings and “grass” denoting the associated weeds. These annotations were compiled into a JSON-formatted file, which was later converted to the Pascal VOC format, producing the CGSSD. Representative images from the CGSSD can be viewed in **Figure 1**.

#### 2.1.2 Data augmentation

**Figure 2:**
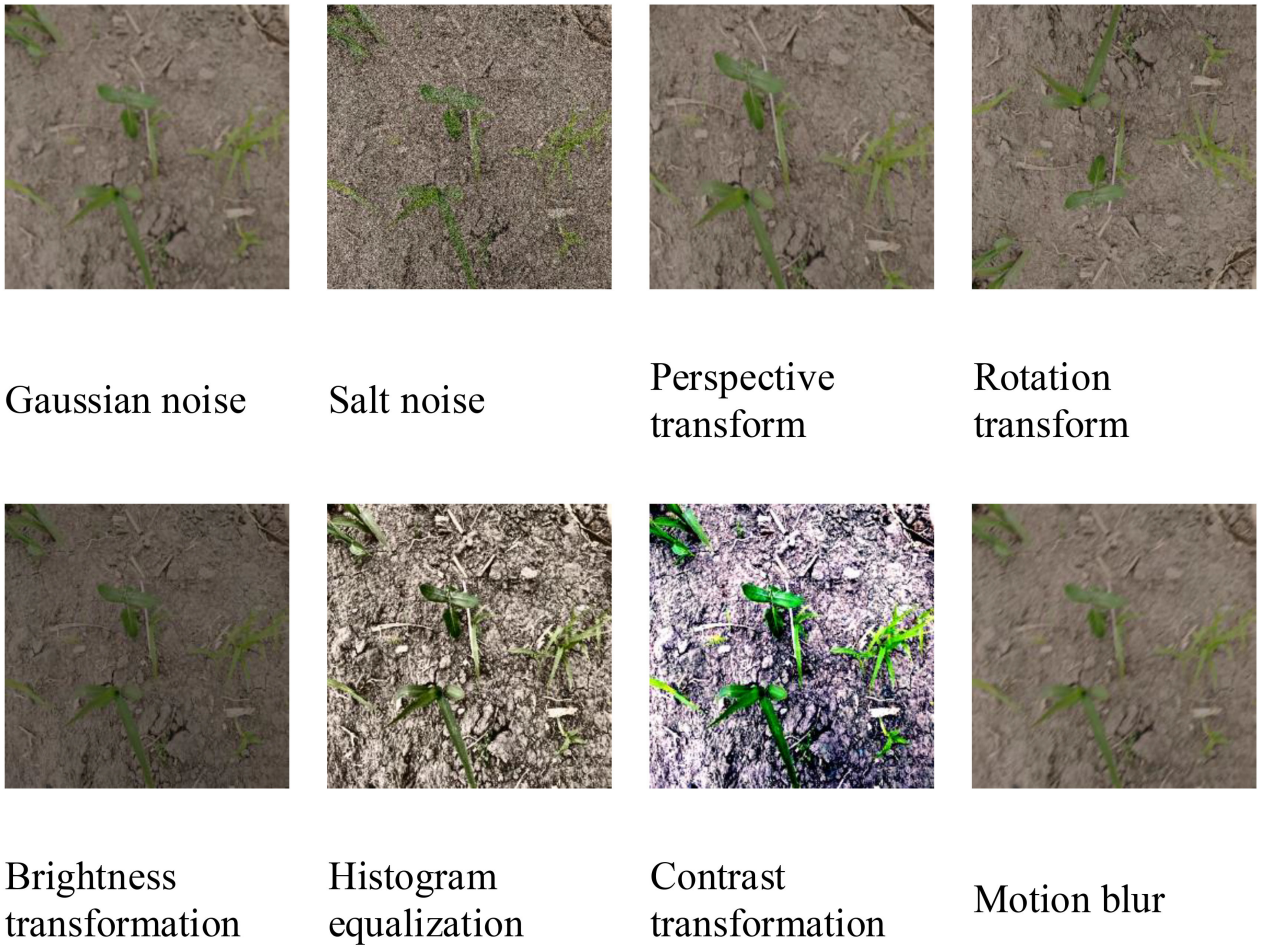
Example of data enhancement samples.

Data enhancement not only increases the number of data samples but also improves their diversity. Thus, our approach reduces the overfitting of certain features, improves the model’s generalization capability, and enhances its robustness in simulating complex field environments. Our enhancement strategies, which are conducted randomly at a 50% probability, include increasing Gaussian noise to simulate conditions such as rain and fog. Random rotation angles are introduced to simulate different camera angles. The color properties, including brightness, contrast, and histogram, were adjusted to represent the different light intensities. Motion blur was incorporated to simulate the blurring effect experienced in field photography. Following the data augmentation strategy described above, the original dataset was augmented to 4000 images using the torchvision.transforms module in PyTorch. We allocated data in an 8:2 ratio for both the training and testing sets, with the training dataset further divided into an 8:2 ratio for the purpose of training and validation. Enhanced data sample visualizations are depicted in **Figure 2**.

#### 2.1.3 Data preprocessing

**Figure 3:**
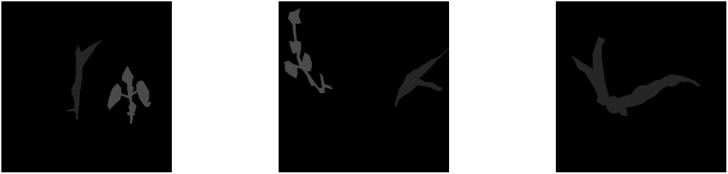
Example of grayscale processing samples.

The annotation of the image is extensive. Color images are converted into black and white (brightness) images, with a grayscale range of 0–255. Converting from color to grayscale not only conserves memory but also expedites the process. Following the grayscale image conversion, the contrast within the images was increased visually to highlight the target area during processing. The processed images are displayed in **Figure 3**.

### 2.2 Experimental environment of semantic segmentation network of field seedlings in maize seedling stage

The technical environment included Windows 10 Professional (64-bit) and was enhanced by software platforms such as Anaconda 3.5.0, CUDA8.2, and CUDNN10.2. The computer was equipped with 16 GB of RAM, powered by an Intel(R) Core(TM) i7-10750H processor, and utilized an NVIDIA GeForce GTX1660Ti graphics card for GPU-intensive tasks. For deep learning target detection, models were constructed within the PyTorch framework. Python served as the primary development language, and the chosen integrated development environment was PyCharm Community Edition 2022.1.3.

### 2.3 Construction of semantic segmentation network of field seedlings in maize seedling stage

#### 2.3.1 General steps for semantic segmentation network construction

The construction of a semantic segmentation network has common stages, such as the encoder, decoder, and training of the semantic segmentation network. By improving the foundation of the U-net semantic segmentation network (Ronneberger and Fischer, 2015), we propose a new semantic segmentation network that obtains better semantic segmentation performance.

**Figure 4:**
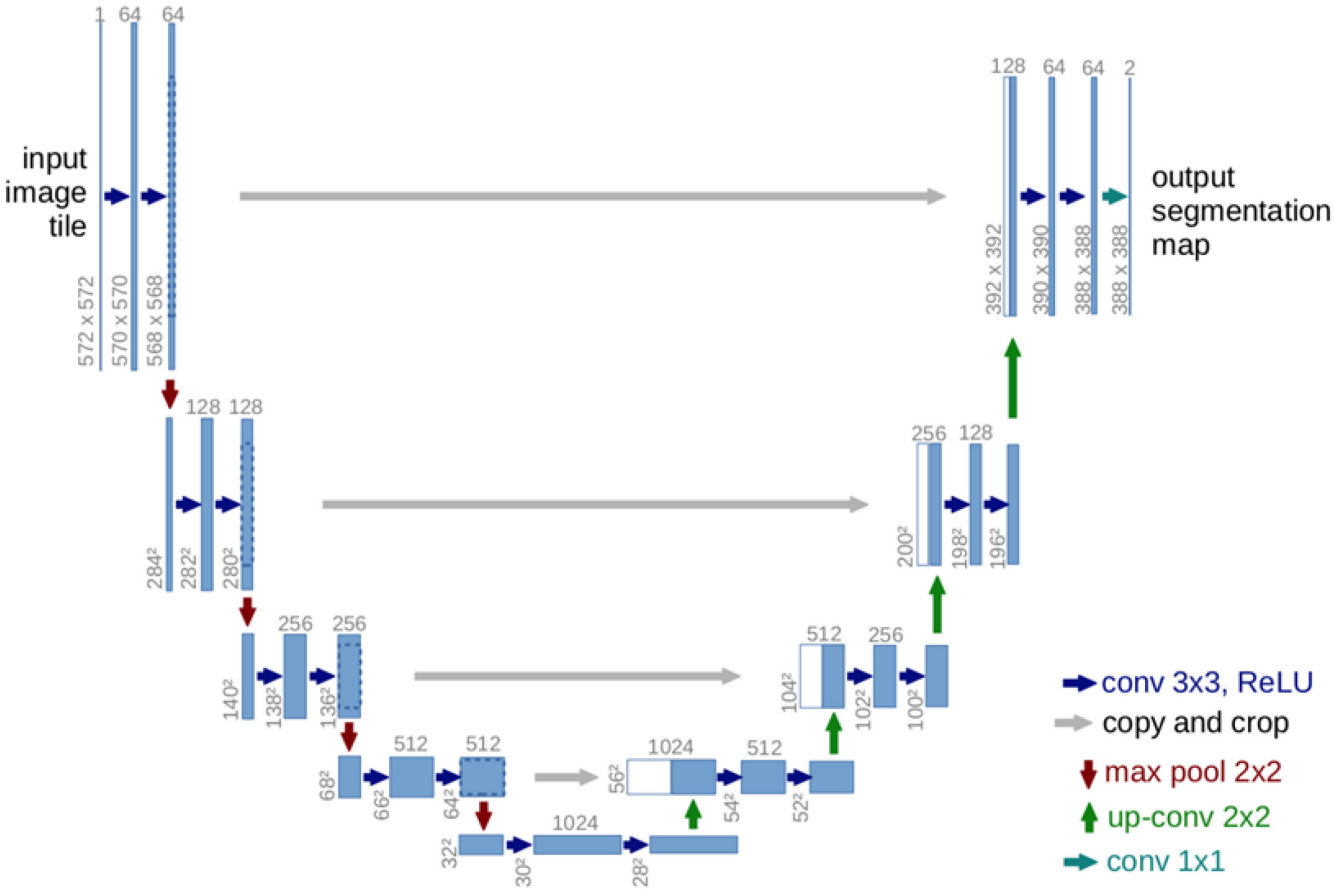
U-net semantic segmentation network structure diagram.

The U-net framework stands out as an essential foundation for recognizing field weeds through semantic segmentation (Mu et al., 2022; Zou et al., 2022; Yang et al., 2023). The structure of the U-net semantic segmentation network, illustrated in **Figure 4**, is symmetrical. The left segment encapsulates the downsampling process or the encoder, while the right side encompasses the upsampling process or the decoder. Within the encoder, each layer consists of two 3 × 3 convolutional kernels, both of which employ the ReLU activation function, followed by a 2 × 2 maximal pooling layer for downsampling, with a pooling layer step size of 2. The feature channels progressively double in number throughout the downsampling process. Within each layer of the decoder, a 2 × 2 convolution is first executed to reduce the number of original feature channels to half. Subsequently, the corresponding cropped feature maps are cascaded, and two 3 × 3 convolution operations, which utilize the ReLU activation function, are performed. Owing to potential losses of boundary pixels during convolution, it is essential to crop the feature maps. The process concludes by mapping each 64-dimensional feature vector to the network’s output layer via a 1 × 1 convolution kernel. The U-net obtains the image’s shallow features through convolution and downsampling in the encoder, whereas it extracts more profound features via convolution and upsampling in the decoder. In the middle, the feature maps from both the encoder and decoder stages are superimposed by overlaying, which refines the image by combining both shallow and deeper features. The final step involves making predictive segmentations based on the obtained feature maps.

#### 2.3.2 Encoder construction

The encoder plays a very important role in semantic segmentation networks and is characterized by its multiple convolutional and pooling layers. Its purpose is to methodically extract features from the input image while compressing the spatial scale of such information. The superior performance of ResNeXt-50 (Xie et al., 2017) in the ResNet (Krizhevsky et al., 2012) series makes our encoder a clear choice in the improved semantic segmentation network. The integration of the residual structure (Krizhevsky et al., 2012) and inception structure (Szegedy et al., 2015) in the ResNeXt network helps avoid issues such as vanishing gradients, all while obtaining better feature extraction at a lower computational cost.

**Figure 5:**
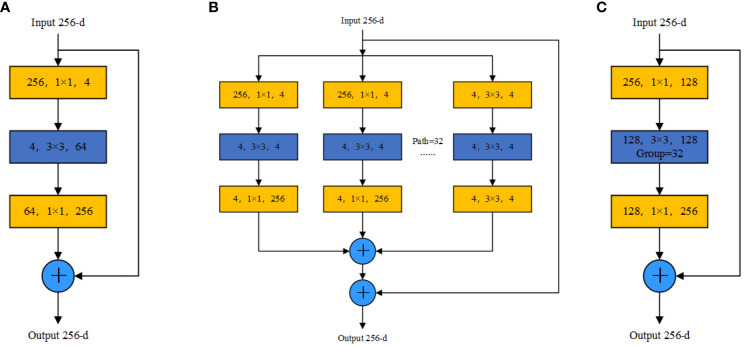
ResNet and ResNeXt block structure diagrams: **(A)** ResNet block structure diagram, **(B)** ResNeXt block structure diagram, and **(C)** ResNeXt block structure equivalent structure diagram.

The conceptual framework of the ResNeXt network (Xie et al., 2017) derives inspiration from the split-transform-merge strategy used in GoogLeNet’s “Inception” module (Szegedy et al., 2015) and further improves upon the ResNet network (Krizhevsky et al., 2012). By melding the “Inception” concept into ResNet’s residual module, a three-layer structure emerges: a 1 × 1 convolution in Layer 1, a 3 × 3 convolution in Layer 2, and another 1 × 1 convolution in Layer 3. The convolutions in Layers 1 and 3 are responsible for dimensionality adjustments, both reducing and enhancing, respectively. This is illustrated in **Figure 5A**). By incorporating the “Inception” concept, ResNeXt’s residual module replaces single-channel convolution with a grouped convolution approach. While the “Inception” module adopts various convolution kernel sizes in each group to obtain features of different scales, enhancing recognition accuracy, the ResNeXt network standardizes the convolution kernel size across groups. This simplification not only streamlines the network’s structure but also improves the accuracy without increasing the parameter complexity. Additionally, the residual module solves potential gradient and degradation problems as the network deepens. Features such as “cardinality,” “sensitivity,” and “density” also find their place in the ResNeXt architecture. In the ResNeXt network, the “cardinality” parameter is introduced to indicate the number of groups. Based on this, the convolutional layers are evenly divided into structures with identical convolutional kernels and input/output channel dimensions. A commonly used structure is the 32 × 4d format, which we have also chosen for our approach. The architecture of the ResNeXt block begins by employing 256 1 × 1 convolution operations to compress the input feature information. The compressed feature map is divided into 32 groups according to the channel dimension, where each group contains four channels. Group convolution then facilitates feature extraction, employing four 3 × 3 convolution operations for every group. The process concludes with each group being expanded using a 1 × 1 convolution. Group convolution has distinct advantages: Each convolution kernel performs only on the input features of its specific group, which reduces the number of convolution operations. Simultaneously, the method of grouping input features ensures that convolution kernels process only the current segment of the feature map at any given time. As a result, the ResNeXt block structure can better extract feature information from each part, promoting diversified feature extraction. This approach not only increases the model’s robustness but also improves its segmentation accuracy. The ResNeXt block structure is illustrated in **Figures 5B, C**).

The ResNeXt model calculation formula is represented by Equation 1:


(1) $$Y=X+\sum_{i=1}^{C}Ti(X)$$

where X represents the input feature and Ti(X) represents any function; in this context, X represents the stacking of three convolution layers. C represents the number of groups.

**Figure 6:**
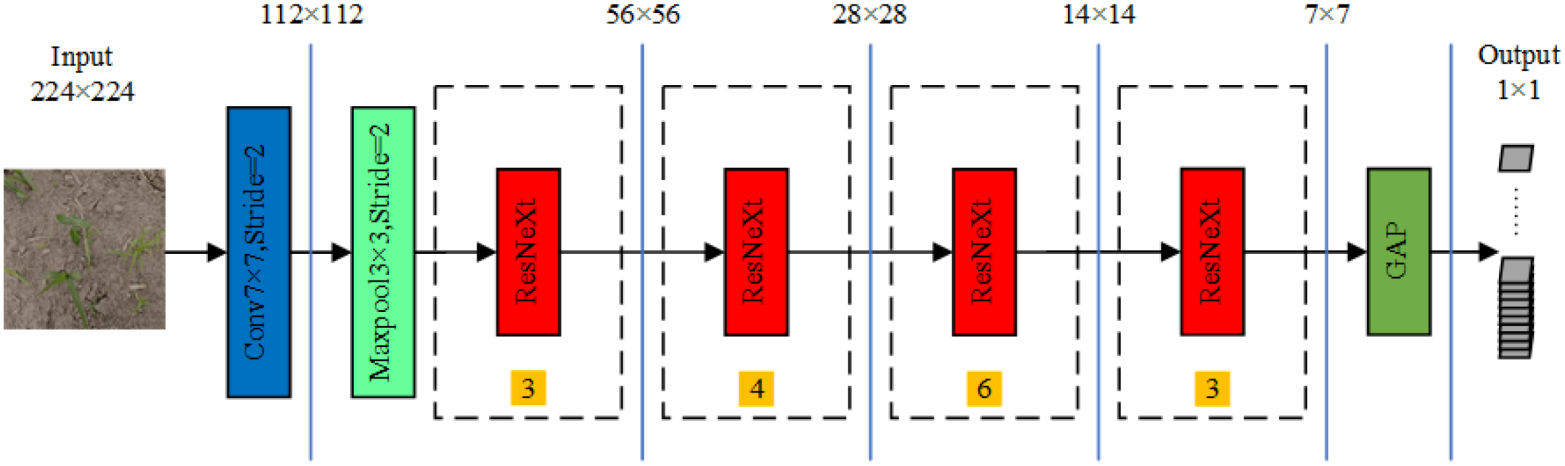
ResNeXt-50 network structure; numbers within yellow squares represent the repetition count of each corresponding module.

The architecture of the ResNeXt-50 network consists of one convolutional layer, four ResNeXt block structures, two pooling layers, and one fully connected layer. The four ResNeXt block structures were reused 3, 4, 6, and 3 times. A depiction of the ResNeXt-50 network is presented in **Figure 6**. The network accepts an input image size of 224 × 224 pixels, which is processed for feature extraction by the ResNeXt-50 network. The specific parameters of the network are enumerated in **Table 1**. The operations in the 2^nd^, 3^rd^, 4^th^, and 5^th^ stages correspond to the operations of the ResNeXt block structure. The postconvolution parameters indicate the convolution kernel size and the number of input channels.

**Table 1:** ResNeXt-50 network parameters.

Layer name	Output shape	Layer type(32×4d)		Repeat
Layer 1	112×112	Conv 7×7 stride=2		—
		Maxpool 3×3 stride=2		
Layer 2	56×56	Conv 1×1 128	C=32	3
		Conv 3×3 128		
		Conv 1×1 256		
Layer 3	28×28	Conv 1×1256	C=32	4
		Conv 3×3 256		
		Conv 1×1 512		
Layer 4	14×14	Conv 1×1 512	C=32	6
		Conv 3×3 512		
		Conv 1×1 1024		
Layer 5	7×7	Conv 1×1 1024	C=32	3
		Conv 3×3 1024		
		Conv 1×1 2048		
—	1×1	GAP		—

#### 2.3.3 Decoder stage

In a semantic segmentation network, the decoder functions opposite to the encoder. It decodes the highly abstracted features in a layered manner to produce a segmentation result. The decoder usually consists of upsampling and convolutional layers. The upsampling layer can also be a transposed or inverse convolutional layer. Its main role is to expand the extracted feature map and decode the extracted abstract features using a convolutional layer. Instead of using traditional convolution, our approach integrates deformable convolution (Dai et al., 2017) with learnable offsets in the decoder stage. This method increases the sensory field, improves the correlation among pixels targeted for segmentation, and enhances the model’s robustness. Additionally, after each decoder layer, we embed the scSE hybrid attention mechanism (Roy et al., 2018). This mechanism effectively learns features from both channel and spatial dimensions, thus eliminating redundant features and improving semantic segmentation accuracy.

**Figure 7:**
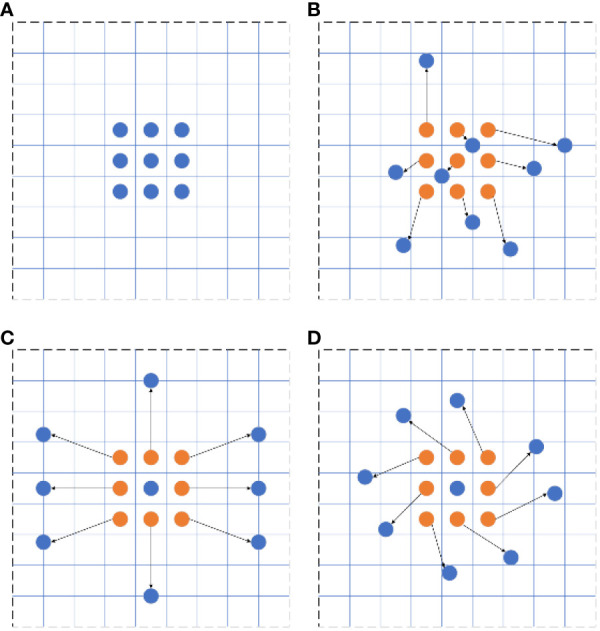
Comparison between standard and deformable convolution sampling points: **(A)** standard convolution, **(B)** deformable convolution, **(C)** deformable convolution special case I, and **(D)** deformable convolution special case II.

Traditional convolutional neural networks employ a fixed convolutional structure for extracting target features, limiting their adaptability and control over various target feature forms. This leads to poor target recognition and generalization. Typically, the convolution kernels in these networks have fixed dimensions (e.g., 3 × 3, 5 × 5), restricting their adaptability to changes in target shapes. To overcome this difficulty, Dai et al. (2017) proposed a deformable convolutional network as a replacement for standard convolution. Their approach increases trainable offsets through the deformable convolutional network, accommodating changes in the target’s shape, thereby improving target detection robustness. Considering that a 1 × 1 deformable convolution does not impact the sensory field and that a 5 × 5 variable convolution demands extensive computation, we chose the 3 × 3 deformable convolution as a replacement for traditional convolution. A comparison of standard and deformable convolution sampling points can be found in **Figure 7**.

In traditional two-dimensional convolution, the output feature map y at local sampling position P_0_ is represented by Equation 2:


(2) $$y(P_{0})=\sum_{P_{n}\in R}w(P_{n})\cdot x(P_{0}+P_{n})$$

where $w(P_{n})$ represents the convolution kernel weight at the sampling location, $x\left(P_{0}+P_{n}\right)$ represents the sampling location of the input feature map, $P_{n}$ represents all the sampling locations within the receptive field, and R represents the receptive field region.

In a deformable convolution, the output feature map y at local sampling position P0 is represented by Equation 3:


(3) $$y(P_{0})=\sum_{Pn\in R}w(P_{n})\cdot x(P_{0}+P_{n}+\text{Δ}P_{n})$$

where {ΔPn∣n=1,N},N=|R| indicates the learnable offset at each sampled position from the standard convolution, usually expressed in decimals. Therefore, the pixel values $x\left(P_{0}+P_{n}+\Delta P_{n}\right)$ at postoffset sampling locations are derived by utilizing bilinear interpolation methods. From the input image, a feature map is extracted using a conventional convolution kernel. Another convolutional layer is then applied to this feature map to obtain the offset for deformable convolution. This results in an output offset of H × W × 2 N, where 2 N means that offsets are obtained in both the x and y directions. During training, the convolution kernels for generating the output feature map and the offsets are learned simultaneously. The offsets are refined through backpropagation using an interpolation algorithm.

The scSE module (Roy et al., 2018) is a combination of channel squeezing and spatial excitation (ssE) and spatial squeezing and channel excitation (csE). The adaptive recalibration of feature maps to enhance useful features while suppressing irrelevant features reduces the computational load of the algorithm and enhances feature extraction. This module assesses the channel–space relationship to extract the importance of input feature maps in both dimensions for the final feature maps. It employs global average pooling for pooling and utilizes sigmoid and ReLU as activation functions.

In this context, 1, 1 × 1 denotes the 1 × 1 convolution for a single channel. The csE module initiates with a global pooling operation on the feature maps, reduces the number of channels to half via a fully connected layer and ReLU activation, recovers the original number of channels using another fully connected layer paired with a sigmoid activation function, and finally multiplies the result with the original feature maps. The csE module recalibrates feature information in the channel dimension. In contrast, the ssE module obtains spatial attention using a 1 × 1 convolution for a single channel followed by a sigmoid activation function and multiplies the result with the original feature map. The output combines the channel-recalibrated ssE with the spatial-recalibrated csE, represented by Equation 4:


(4) XscSE=XcsE+XssE

**Figure 8:**
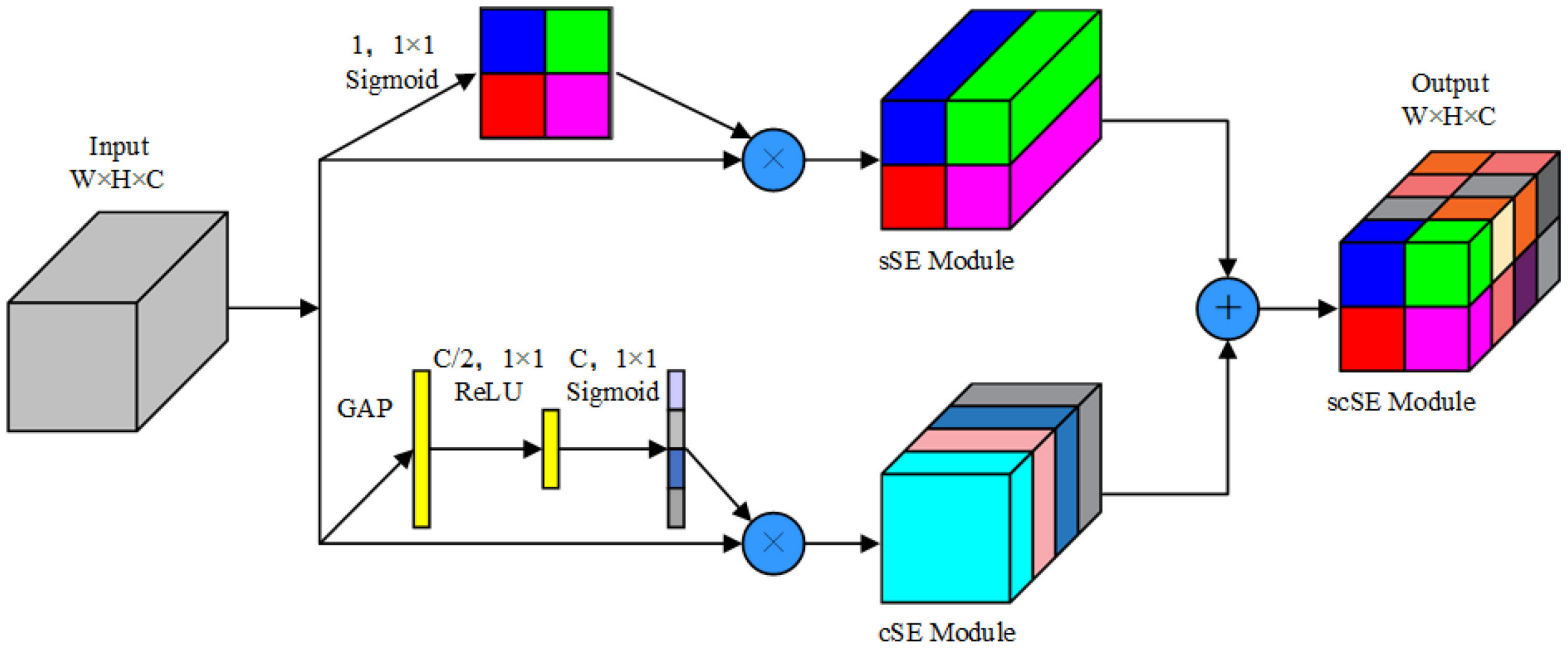
scSE structure.

The output feature diagram highlights important channel and spatial features, refining the neural network’s recognition capabilities by enhancing useful features and inhibiting irrelevant features. This structure is depicted in **Figure 8**.

#### 2.3.4 Overall structure of modified U-net semantic segmentation network

**Figure 9:**
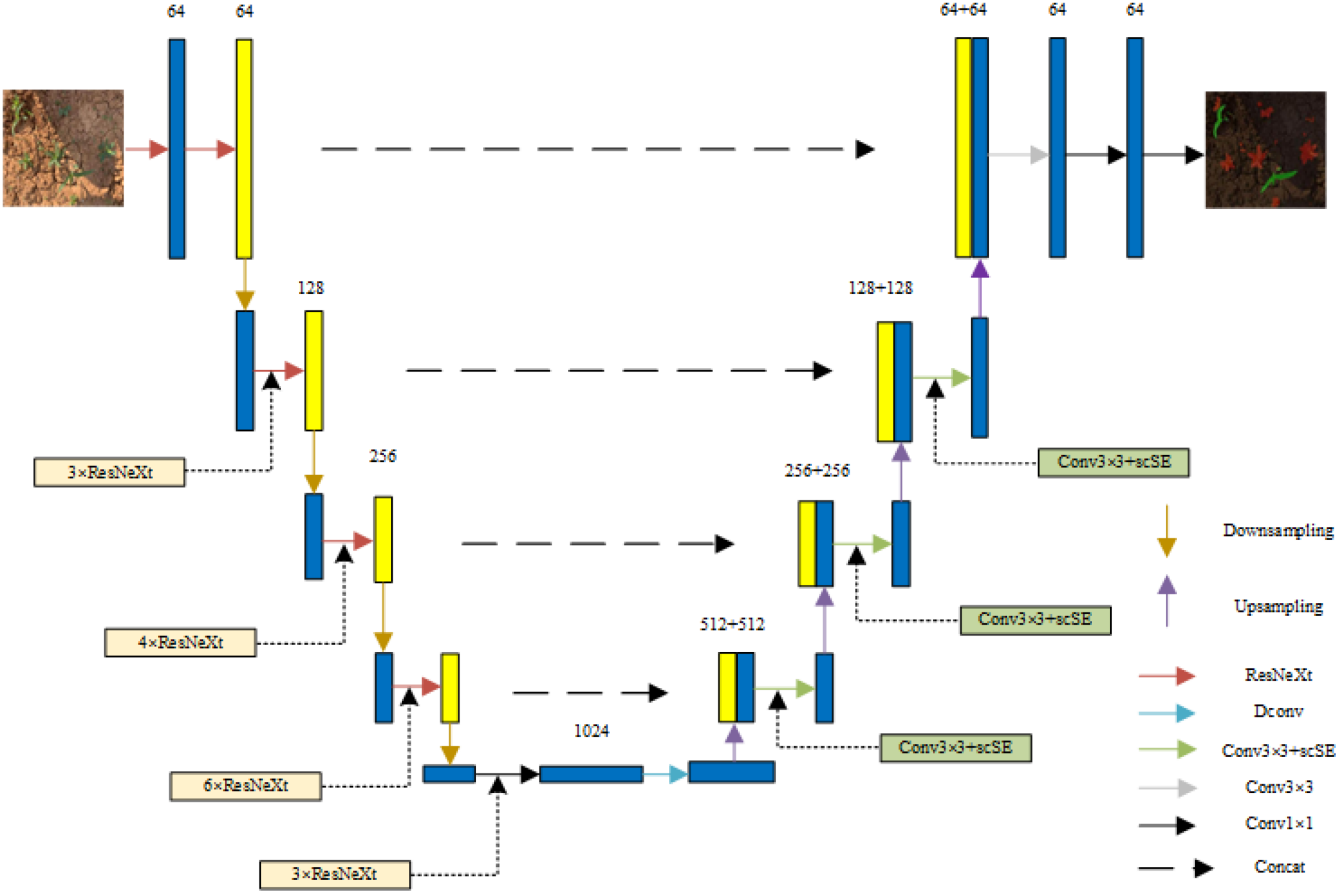
Structure diagram of RDS_Unet.

Our improved U-net semantic segmentation network contains two main parts: an encoder and a decoder. In the encoder stage, we used the ResNeXt-50 network as a replacement for the original backbone feature extraction network. This decision, based on the ResNeXt-50 network’s superior feature extraction capabilities, is particularly evident when analyzing maize field seedlings and grassy images during the seedling stage. Its progress is especially noticeable when overlapping seedlings and grass or when addressing miniscule seedlings and grass targets are present. For the decoder stage, we integrated the scSE attention mechanism module directly after the common 3 × 3 convolution during the 2^nd^, 3^rd^, and 4^th^ upsampling layers. This scSE module facilitates the automation excitation or suppression of valid and invalid features across both channels and spatial dimensions. We also replaced the ordinary 3 × 3 convolution in the first layer of the decoder with a 3 × 3 deformable convolution. This inclusion, with its learnable offsets, increases the deep feature receptive field, improving the correlation among the features slated for segmentation. This ensures that the features encapsulate a certain degree of global characterization. In addition, to avoid any potential loss of seedling image information after upsampling, we implemented a jump layer to fuse the upsampled feature maps with the corresponding encoder feature maps of matching resolution. The final predictive segmentation was executed based on these obtained feature maps. We named this improved U-net semantic segmentation network ResNeXt Dconv scSE_U-Net (RDS_Unet). The structure of the RDS_Unet semantic segmentation network is depicted in **Figure 9**.

#### 2.3.5 RDS_Unet semantic segmentation network training

The encoder and decoder architectures of the RDS_Unet semantic segmentation network were trained via random initialization. The number of iterations was set at 100, with a batch size of 4. The weight parameter commenced with a learning rate of 0.001, the momentum was set at 0.9, and the decay rate was marked at 1e-4. Adam served as the optimizer, and Dice loss was utilized as the loss function.

### 2.4 Evaluating indicators

To evaluate the performance and effectiveness of our proposed semantic segmentation network, we exposed the experimental results to both subjective and objective evaluations. The subjective evaluation involved visually comparing the seedling grass recognition in maize fields during the seedling stage using the segmentation image results. For objective evaluation, we adopted widely recognized metrics in the field of semantic segmentation: the mean intersection over union ratio (MIoU), recall rate (recall), and precision rate (precision). These metrics serve as benchmarks for assessing segmentation performance. These equations are calculated via Equations 5-7:


(5) $$MIoU=\frac{1}{k+1}\sum_{i=0}^{k}\frac{TP}{FN+FP+TP}$$

(6) $$\text{Recall}=\frac{TP}{TP+FN}$$

(7) $$Precision=\frac{\text{TP}}{\text{TP}+\text{FP}}$$

where TP stands for true positives, where a positive class is correctly predicted as positive. FN signifies false negatives, where a positive class is incorrectly predicted as negative. FPs represent false positives, where a negative class is mistakenly predicted as positive. Finally, TN denotes true negatives, where a negative class is correctly predicted as negative.

Furthermore, we introduced frames per second (FPS) as an index to measure the processing speed of the semantic segmentation network.

## 3 Results and analysis

### 3.1 Comparison experiments

To verify the effectiveness of our proposed RDS_Unet semantic segmentation network in recognizing seedling grass during the corn seedling stage in field environments, we chose to compare it against classical semantic segmentation networks such as U-net, PspNet (Zhao et al., 2017), and DeeplabV3 (Chen et al., 2017). We used the CGSSD dataset, which was specifically curated for seedling grass semantic segmentation during the maize seedling stage in the field. We compared the networks based on the MIoU, precision, recall, and FPS metrics. All the networks were trained for 100 iterations without leveraging the pretraining weights of migration learning. The average experimental results obtained from 10 repeated experiments are shown in **Table 2**.

**Table 2:** Experimental results on the CGSSD test set.

Segmentation method	MIoU%	Precision %	Recall%	FPS
U-net	76.45%	87.86%	83.96%	13.8
Pspnet	71.64%	83.17%	79.70%	19.2
DeeplabV3	76.50%	75.61%	70.25%	**24.7**
RDS_Unet	**82.36%**	**91.36%**	**89.45%**	12.6

The bolded font represents the best performance.

**Table 2** clearly shows that our RDS_Unet semantic segmentation network outperforms classical models such as U-net, PspNet, and DeeplabV3 on the self-constructed CGSSD dataset, which focuses on the semantic segmentation of corn seedling stage field seedlings. In particular, compared to U-net, our model improved the MIoU by 5.91%, the precision by 3.50%, and the recall by 5.49%. The unsatisfactory performance of other classical networks can be attributed to the challenging nature of the images in our self-constructed CGSSD dataset. In terms of the semantic segmentation network testing speed, measured in FPS, RDS_Unet matched the U-net’s speed even with the integration of the more complex ResNeXt as its feature extraction backbone. This makes it optimal for placement on various embedded devices, such as Jetson NX, IMX8PLUS, and Raspberry Pi. In summary, the improvements in the RDS_Unet semantic segmentation network prove effective, emphasizing its suitability for recognizing seedlings and grasses during the seedling stage in complex natural environments, balancing both segmentation accuracy and testing speed.

**Figure 10:**
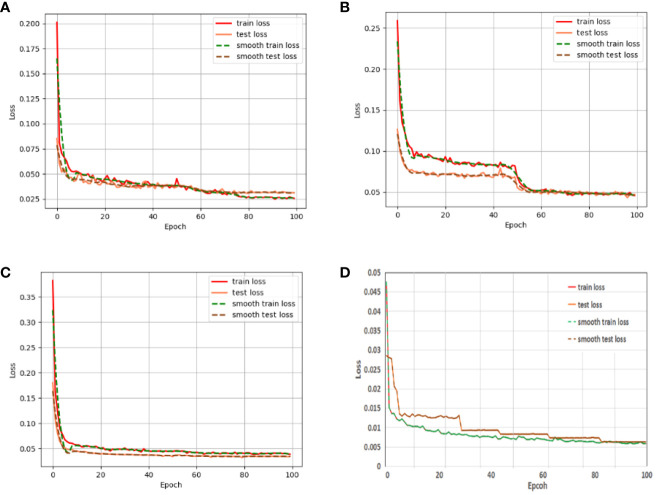
Loss variations on the CGSSD training and testing sets for **(A)** U-net, **(B)** PspNet, **(C)** DeeplabV3, and **(D)** RDS_Unet Note: Green and red indicate corn and weeds, respectively.

**Figure 10** shows the training and testing loss curves of our proposed RDS_Unet semantic segmentation network compared with those of the classical semantic segmentation networks U-net, PspNet, and DeeplabV3. The figure demonstrates that none of the semantic segmentation networks displayed an overfitting phenomenon. Additionally, our RDS_Unet had smaller training and testing losses, faster convergence speed, and better model robustness than did the classical semantic segmentation networks. After 100 iterations, the training and testing losses of the classical semantic segmentation networks stabilized. However, the losses for our RDS_Unet did not stabilize, indicating that the model has significant room for improvement.

**Figure 11:**
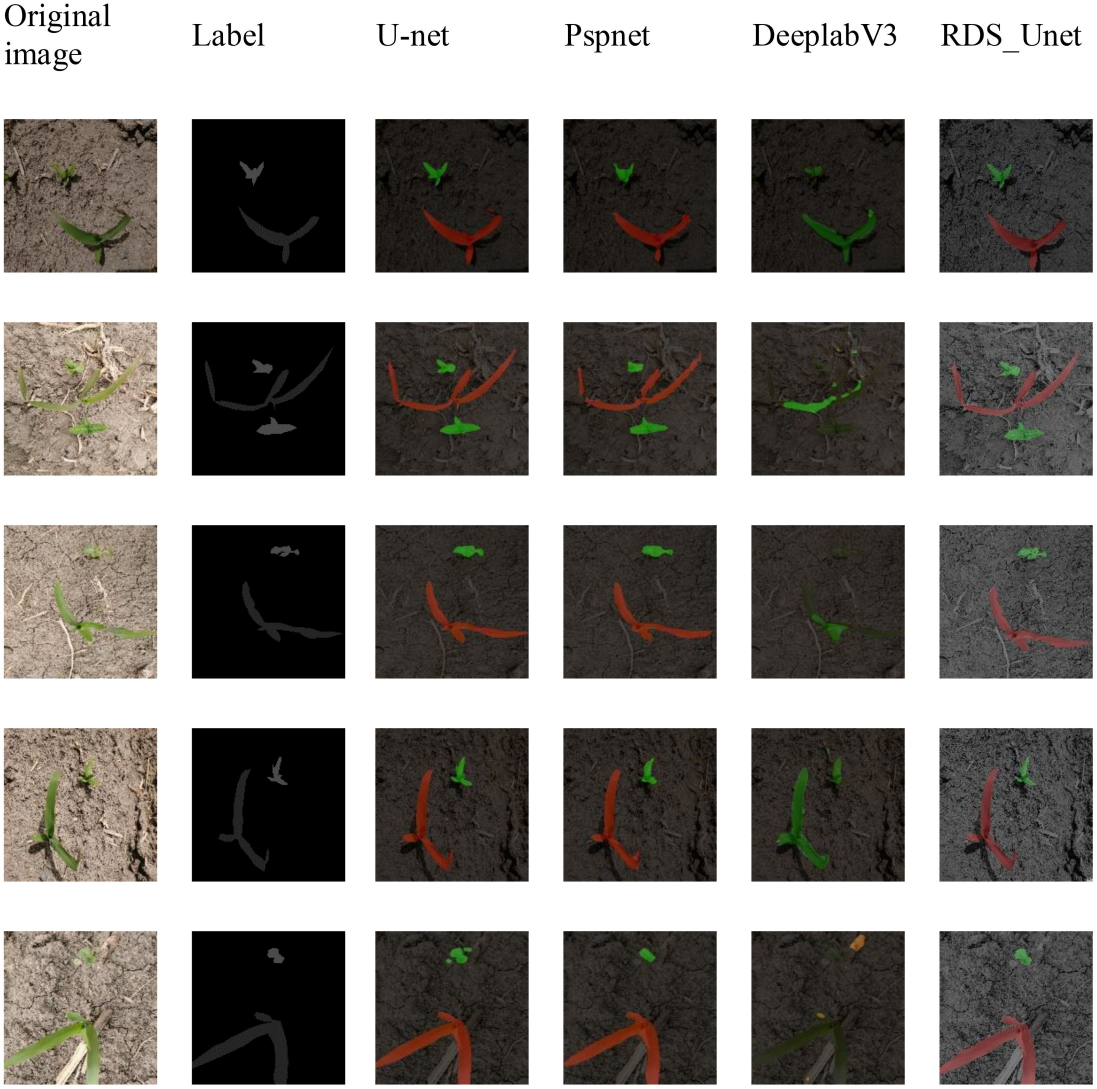
Visual comparative analysis of semantic segmentation performance on the CGSSD test dataset Note: Green and red indicate corn and weeds, respectively.

**Figure 11** presents a comparison between our proposed RDS_Unet semantic segmentation network and the classical models in the context of seedling grass semantic segmentation during the maize seedling stage. The figure indicates that the DeeplabV3 network exhibits pronounced misclassification when segmenting corn seedlings and field companion weeds. In contrast, the U-net and PspNet networks sometimes present unclear boundaries in the segmentation of smaller target weeds within the same context. Therefore, our RDS_Unet demonstrates a significant advantage in addressing both the segmentation-type errors and the unclear boundaries observed when segmenting corn seedlings and companion weeds.

### 3.2 Ablation experiment

To further evaluate the effect of our proposed improvement scheme on the U-net segmentation network’s performance, we conducted an ablation experiment using our self-constructed dataset, CGSSD. In this ablation experiment, the U-net network served as the foundation. Specifically, we evaluated the impacts of (i) replacing the original U-net backbone feature extraction network with ResNeXt in the encoder, (ii) replacing the traditional convolution for a deformable convolution with learnable offsets in the decoder, and (iii) introducing the scSE hybrid attention mechanism after the upsampling layer in the decoder. The impact on the segmentation performance of the U-net semantic segmentation network. The average results of the ablation experiments obtained from 10 repeated experiments are presented in **Table 3**.

**Table 3:** Results of disruption experiments on the CGSSD test set.

Segmentation method	MIoU%	Precision %	Recall%
U-net	76.45%	87.86%	83.96%
U-net+①	78.69%	88.62%	85.14%
U-net+①+②	79.47%	89.75%	86.24%
U-net+①+②+③	**82.36%**	**91.36%**	**89.10%**

①ResNeXt-50, ②Deformable Convolution, ③ scSE, bold for best performance.

**Table 3** shows that when the U-net+① semantic segmentation network is compared with the U-net semantic segmentation network, the adoption of the ResNeXt-50 network as the backbone feature extraction network in the encoder stage significantly improves the network’s ability to extract features from overlapping occlusion targets and small-target seedlings and grasses. This approach results in an enhancement in the network’s semantic segmentation performance by 2.24% in MIoU, 0.76% in precision, and 1.18% in recall. When comparing the semantic segmentation network of U-net+①+② with that of U-net+①, the adoption of deformable convolution with learnable offsets instead of the traditional convolution in Layer 1 of the decoder stage makes the U-net+①+② network more effective than U-net+①. The traditional convolution scheme in Layer 1 of the decoder stage increases the sensitivity of the semantic segmentation network to changes in the shape of the field seedling target. This leads to an improvement in the network’s semantic segmentation performance by 0.78% in MIoU, 1.13% in precision, and 1.1% in recall. Furthermore, the U-net+①+②+③ semantic segmentation network displays greater sensitivity to shape changes in field-generated seedlings and grasses than does the U-net+①+② network. This is attributed to the introduction of the scSE hybrid attention mechanism after the conventional convolution in the 2^nd^, 3^rd^, and 4^th^ upsampling layers of the decoder stage. With this mechanism, the feature extraction capability is further improved. As a result, the network’s semantic segmentation performance improved the MIoU by 2.89%, the precision by 1.61%, and the recall by 2.86%.

**Figure 12:**
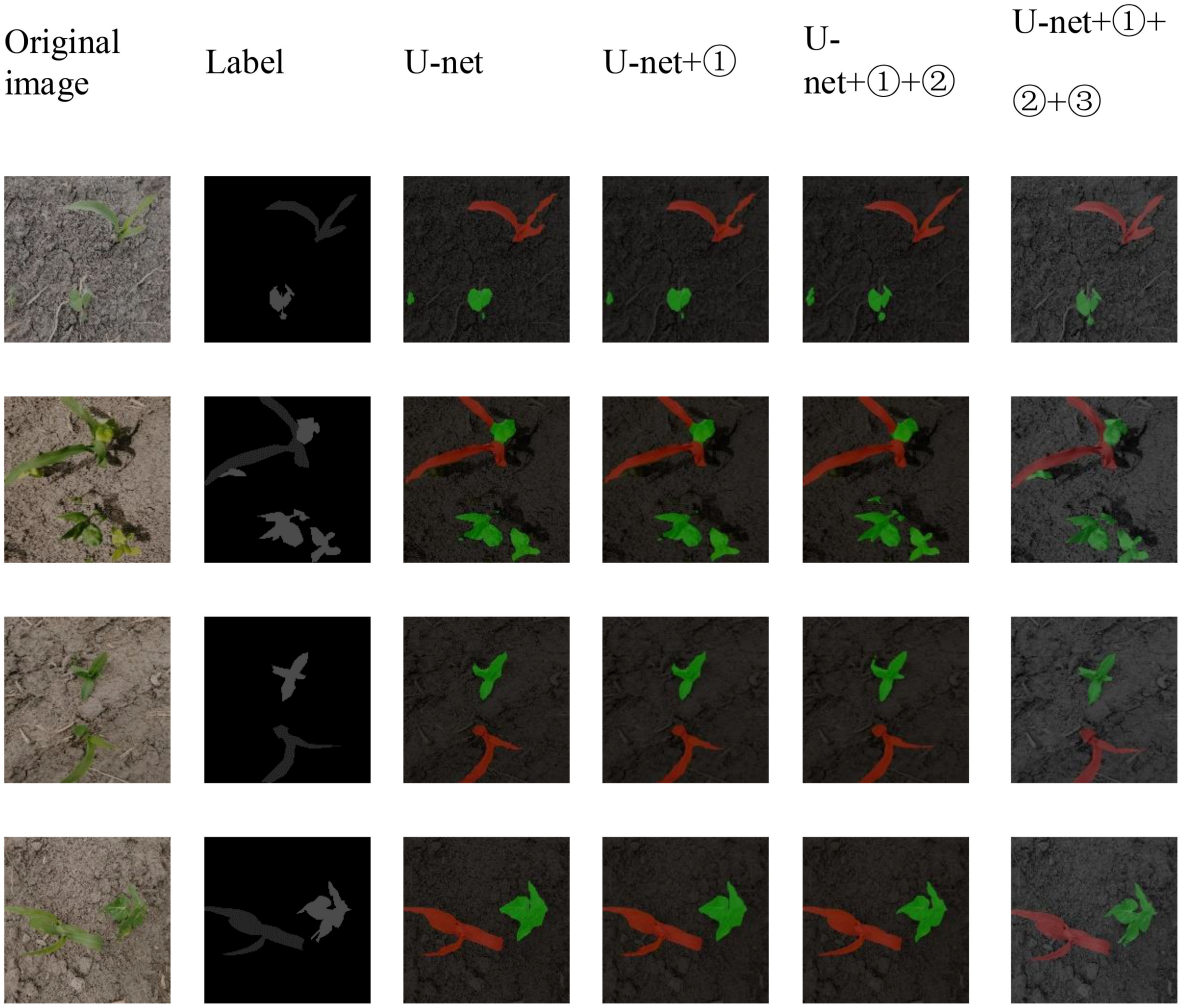
Visual comparison of the semantic segmentation effects in disintegration experiments on the CGSSD test set: ① ResNeXt, ② Deformable Conv, and ③ scSE.

**Figure 12** displays the visualization of the semantic segmentation effects achieved by the semantic segmentation network for each improvement scheme in the ablation experiment. Visualization of the segmentation effects of the U-net semantic segmentation network clearly reveals that this network has limitations in accurately classifying miniscule seedling grass targets and maintaining clear segmentation boundaries. Observing the U-net+① semantic segmentation network, it becomes clear that these network issues, when compared to those of the basic U-net regarding classification accuracy and segmentation boundary clarity, remain largely unresolved. When examining the U-net+1 semantic segmentation network, similar challenges in terms of classification accuracy and boundary definition persist, especially when classifying tiny seedling grass targets. Misclassifications and ambiguous segmentation of boundaries still exist. This shows that replacing only the backbone feature extraction network, despite its capacity to enhance semantic segmentation performance test results, does not result in significant advancements, making subsequent ② and ③ improvements necessary. A review of the U-net+①+② segmentation images reveals that although a discernible enhancement is observed in the clarity of the seedling grass segmentation boundaries, the phenomenon of tiny seedling grass target misclassification still occurs. The improvement in the fuzzy boundary of seedling segmentation verifies the effectiveness of using deformable convolution with learnable offset in place of traditional convolution. Deformable convolution expands the sensing field, increasing the correlation among the pixels to be segmented and leading to sharper segmentation boundaries. After evaluating the U-net+①+②+③ segmentation, the results are markedly superior to those of previous studies, demonstrating its effectiveness. The visualization diagram shows that this model achieved significant improvements in terms of classification accuracy and segmentation boundary sensitivity for tiny seedling targets, especially for overlapping and occluded regions. The significant improvement in the semantic segmentation performance verifies the effectiveness of the introduction of the scSE hybrid attention mechanism. The strength of the scSE mechanism lies not only in its ability to learn features from both channel and spatial dimensions but also in its ability to inhibit redundant features by reducing the weights of invalid features. This suppression of invalid and redundant features is crucial for substantially elevating the quality of semantic segmentation. These results underscore the conclusion that simply improving the feature extraction capability leads to limited improvement in semantic segmentation, as initially proposed in the ① improvement scheme.

## 4 Discussion

### 4.1 Purpose of intelligent seedling recognition research

The importance of weed identification during the seedling stage in agriculture is self-evident. For healthy growth of crop plants, it is imperative to detect and remove weeds promptly and accurately. However, the complexity of the field environment, characterized by overlapping seedlings, grass, and shadows, coupled with the striking resemblance between certain grassy weeds and maize seedlings during the seedling phase, complicates the accurate identification of maize and associated weeds. This makes their precise identification quite challenging. In response to this practical challenge, we propose the RDS_Unet semantic segmentation network, which is tailored for precise seedling weed recognition in the field during the maize seedling stage and yields better segmentation results. This network offers technological support for the automated recognition of seedling weeds via intelligent mechanical weeding equipment. Such advancements can hinder weed proliferation, reduce reliance on agro-chemical herbicides, reduce the detrimental impacts of weeds on crop yields, and realize sustainable agricultural production.

### 4.2 Necessity of semantic segmentation network model improvement

The effectiveness of semantic segmentation networks, particularly the U-net model, in field seedling recognition has been well proven, making them a prime choice for further research advancements (Amin et al., 2022; Yu et al., 2022; Ma et al., 2023). The U-net semantic segmentation network is distinguished by its nearly symmetrical U-shaped structure comprising an encoder and a decoder. The encoder’s backbone feature network needs to possess strong image feature abstraction capabilities, while the decoder’s upsampling layers need to excel in parsing these abstracted image features. Given that our self-constructed dataset for the corn seedling stage, CGSSD, is used with complex images showcasing overlapping seedlings, occlusions, and tiny seedling targets that pose segmentation challenges, the requirement for an adept backbone feature extraction network is higher. While both semantic segmentation and image classification are different computer vision tasks, their demands for feature extraction capabilities remain consistent. In this study, we chose the ResNeXt-50 network, which is renowned for its outstanding performance in image classification, as the backbone feature extraction network for our improved U-net semantic segmentation. The residual structure in ResNeXt, coupled with its inception structure, effectively avoids gradient vanishing while achieving diverse feature extraction at a low computational cost. This approach is beneficial for accessing difficult-to-segment images in our self-constructed dataset. In addition, semantic segmentation tasks aim to classify input images on a pixel-by-pixel basis. The correlations among these pixels, especially in challenging segmentation scenarios, are important and should not be ignored. As a result, instead of conventional convolution, we deployed deformable convolution with a learnable offset in the decoder’s first layer. This approach enables the discovery of correlations between pixels over a larger field, aligning well with the requirements of our self-constructed dataset. Deformable convolution is able to explore the correlation between the pixels to be segmented with a larger sensory field, which is also useful for hard-to-segment sample images in our self-constructed dataset. The strength of a semantic segmentation system depends not only on strong image feature abstraction but also, importantly, on its ability to analyze these abstract features during the decoding stage. Deeper backbone feature extraction networks, while capturing essential features, also inevitably introduce some invalid, redundant feature information. Such invalid information can often be the main cause of misclassification and unclear segmentation boundaries. Therefore, we adopted the scSE hybrid attention mechanism after ordinary convolution in the 2^nd^, 3^rd^, and 4^th^ layers of the decoder stage. This attention mechanism dynamically emphasizes effective features while suppressing ineffective features across both channel and spatial dimensions. Combined with our study, the encoder, utilizing the ResNeXt-50 network, harbors a large amount of abstract feature information from the images. Thus, the data sent to the decoder via the jump layer inevitably comprise invalid and redundant information. By employing the scSE hybrid attention mechanism, we suppress these inefficiencies, improving the segmentation performance of our proposed RDS_Unet network. Notably, CNNs inherently function as “black boxes.” The absence of a standard blueprint when selecting network structures often prevents the combination of an encoder and decoder structure from resulting in a good CNN. Nevertheless, our research obtained a semantic segmentation network model that demonstrated good performance. Compared to the original Unet model, our proposed improved semantic segmentation network model, RDS_Unet, introduces a ResNeXt-50 backbone feature extraction network, deformable convolutions, and the scSE attention mechanism. While these enhancements lead to improved model recognition accuracy (a 3.50% increase), they also come with additional energy requirements and time consumption. However, unlike those of other intelligent plant protection systems, the performance of intelligent mechanical weeding systems relies more on the model’s recognition accuracy. When other intelligent plant protection systems have deficiencies in terms of recognition accuracy, they may only result in suboptimal effects, such as overspraying or underspraying chemical pesticides. In contrast, an intelligent mechanical weeding system with low recognition accuracy poses a risk of causing damage to crops, leading to crop yield losses. Therefore, considering various factors, it is beneficial to judiciously increase energy requirements and time consumption to achieve higher recognition accuracy in the context of intelligent mechanical weeding systems.

### 4.3 Comparative analysis of similar studies

Guo et al. (2023) proposed a weed recognition model based on the UNet network model and the Vision Transformer (ViT) classification algorithm. Their study focused on corn, wheat seedlings, and field weeds. Initially, they used the UNet model for image segmentation, followed by extracting features of segmented green plant leaves using the PIL algorithm from the Python Imaging Library. Finally, the image features were input into the Vision Transformer model to recognize and classify field corn, wheat seedlings, and weeds. Although their combined recognition model achieved an accuracy of 99.3% on a self-built dataset, compared to our proposed RDS_Unet model, their combined model’s output included only image classification results. It is known that image classification results do not contain positional information about the targets to be identified in the image, which is a limitation. Moreover, the ViT classification algorithm model has a large volume and many parameters, leading to a significant consumption of computational resources and a relatively slow recognition speed. Yu et al. (2022) introduced a semantic segmentation method that combines an improved UNet structure and an embedded channel attention mechanism (SE) module. Their research focused on soybeans, grassy weeds, and broadleaf weeds in complex field environments. They used ResNet34 with an embedded channel attention mechanism (SE) module as the backbone feature extraction network in the encoder stage. The features obtained from the first 34 downsampling tasks were restored to the same original image by combining the feature maps through deconvolution layers as an upsampling method, along with low-dimensional feature maps across connections. Although their combined recognition model achieved an accuracy of 96.11% on a self-built dataset, compared to our proposed RDS_Unet model, even though both models used the ResNet network structure as the backbone feature extraction network in the encoder, the ResNeXt with the “Inception” structure in our model has stronger feature extraction capabilities. Additionally, we employed deformable convolutions to enhance the correlation between each pixel to be segmented, improving the robustness of our model. Finally, when attention modules are used, only channel attention is focused on, while spatial attention is also an indispensable factor in semantic segmentation tasks.

### 4.4 Limitations of this study

Constrained by the limitations of the current technological platform for autonomous agricultural driving based on computer vision technology (Xie et al., 2023), the proposed RDS_Unet semantic segmentation network focuses only on segmenting corn seedlings and accompanying weeds in a corn field without incorporating land information. Therefore, this network is applicable to manned mechanical weeding equipment and is not equipped for route planning.

## 5 Conclusion

We collected seedling and weed images using ball camera equipment during the maize seedling stage and created the maize seedling and weed semantic segmentation dataset CGSSD. These data were further augmented by simulating complex weather conditions in the field. Building on this, we introduce an improved RDS_Unet semantic segmentation network tailored for seedling grass image segmentation during the maize seedling stage. This network accurately segments maize seedlings and accompanying weeds in maize fields. When trained on the CGSSD dataset for only 100 rounds, the network achieved a test precision of 91.36% and a test speed FPS of 12.6. While the RDS_Unet network’s speed may be slightly subpar, its precision surpasses that of other classical semantic segmentation networks. This advancement provides valuable technical support for smart mechanical weeding equipment designed for automatic seedling grass recognition.

In the future, we will focus on building a practical mechanical weeding system. This system will encompass components such as the seedling vision recognition system, communication protocols and modules, map construction and path planning software, remote monitoring and management systems, data storage and analysis, safety and fault recovery systems, and software components for software updates and maintenance tools. The seedling vision recognition system is a core functional module in this system. We aim to optimize the recognition accuracy and speed of the RDS_Unet semantic segmentation network through techniques such as transfer learning and lightweight approaches (model pruning, and weight quantization). This optimization will facilitate better compatibility with the operational requirements of various types of embedded devices.

## Data availability statement

The original contributions presented in the study are included in the article/supplementary material. Further inquiries can be directed to the corresponding author.

## Author contributions

JC: Methodology, Writing – original draft. FT: Conceptualization, Funding acquisition, Writing – review & editing. NB: Software, Writing – original draft. YF: Visualization, Writing – original draft.
